# Low birth weight in São Luís, northeastern Brazil: trends and associated factors

**DOI:** 10.1186/1471-2393-14-155

**Published:** 2014-05-01

**Authors:** Helma Jane Ferreira Veloso, Antônio Augusto Moura da Silva, Heloísa Bettiol, Marcelo Zubarán Goldani, Fernando Lamy Filho, Vanda Maria Ferreira Simões, Rosângela Fernandes Lucena Batista, Marco Antônio Barbieri

**Affiliations:** 1 Departamento de Saúde Pública, Universidade Federal do Maranhão, Rua Barão de Itapary 155 Centro, São Luís, MA 65020-070, Brazil; 2 Departamento de Puericultura e Pediatria. Faculdade de Medicina de Ribeirão Preto, Universidade de São Paulo. Hospital das Clínicas de Ribeirão Preto, 7°. andar. Av. Bandeirantes, 3900, Ribeirão Preto, SP 14049-900, Brazil; 3 Departamento de Pediatria e Puericultura. Faculdade de Medicina, Universidade Federal do Rio Grande do Sul. Rua Ramiro Barcelos, 2400, Porto Alegre, RS 90035-003, Brazil

**Keywords:** Low birth weight, Preterm birth, Intrauterine growth restriction, Cohort studies

## Abstract

**Background:**

To analyze trends in LBW (low birth weight) rate using birth registry data and identify factors associated with LBW in São Luís comparing two birth cohorts separated by a 12-year interval.

**Methods:**

2,426 births were included in 1997/98 and 5,040 in 2010. The dependent variable was LBW (<2,500 g). Multiple logistic regression was performed to determine the association of independent variables with LBW. Data were also obtained from SINASC (Brazilian National Birth Registry) to analyze stillbirth and LBW rates trends from 1996 to 2010, using 3-year moving averages.

**Results:**

LBW, intrauterine growth restriction (IUGR) and preterm birth rates did not differ between the two cohorts. Despite this, birth registry data showed increasing LBW rate up to 2001, coinciding with decreasing stillbirth rate. Both stillbirth and LBW rates decreased thereafter. A significant reduction was observed in the percentage of teenage mothers, mothers with up to 4 years of education, family income up to one minimum wage and mothers who did not attend prenatal care. There was an increase in maternal age ≥35 years and schooling ≥12 years. The variables associated with LBW in 1997/98 were young maternal age (<18 years), maternal smoking during pregnancy and primiparity. Variables that remained in the adjusted model in 2010 were female gender, income <3 minimum wages, lack of prenatal care, maternal smoking during pregnancy and primiparity.

**Conclusions:**

Although LBW rate did not differ between the two cohorts, this apparent stability masked an increase up to 2001 and a decrease thereafter. The rise in LBW rate paralleled reduction in the stillbirth rate, suggesting improvement in obstetrical and newborn care. Maternal, socioeconomic and demographic factors associated with LBW differed between the two cohorts, except for smoking during pregnancy and parity that were significantly associated with LBW in both cohorts.

## Background

Considered to be the major factor affecting perinatal, neonatal and postnatal health, low birth weight (LBW) is defined as a birth weight of less than 2,500 g [1].

LBW is due either to preterm birth, most common in developed countries, to intrauterine growth restriction (IUGR), more frequent in developing countries or to a combination of both. Factors associated with LBW are low maternal weight and height, multiple births, low calorie intake, hypertension during pregnancy, maternal smoking, genetic syndromes, hard maternal physical work, maternal exposure to toxic substances, and inadequate prenatal care use [2,3].

LBW rates differ in various regions of the world, being higher in less developed countries, since they are associated with unfavorable socioeconomic conditions. LBW rate is estimated to be 15% for developing countries and 7% for developed countries [4,5]. LBW rate was 9.1% for Brazil in 2010 and 9.6% for São Luís in 1997/98 [5,6].

Increasing LBW rates began to be observed in developed countries in the 1980 decade [7]. The increase in LBW rate was observed in the United States and was attributed to increasing preterm and multiple births [8]. Increasing preterm and LBW rates were also observed in Canada, explained in part by the increase in twin births [9]. Finland and France were the only developed countries that reported reduction in preterm birth rate and increase in LBW rate [10,11].

In Brazil, LBW rate increased significantly in its state capital cities from 8.5% in 1996 to 9.1% in 2010. Part of this increase in LBW rate may be explained by a simultaneous increase in the multiple birth rate and in the number of newborns (NB) weighing 500 to 999 g and by a reduction in the stillbirth rate [6].

Studies monitoring LBW trends as well as changes in its risk factors over time are important in order to determine whether such factors are changing, thus allowing identification of targets for public health interventions.

Increasing LBW rate was also observed in Brazilian birth cohorts performed in Ribeirão Preto and Pelotas. In Ribeirão Preto, part of the increase in LBW rate was attributed to shortened gestational age due to elective cesarean sections [12]. In Pelotas, the increase in LBW rate was explained by rising preterm and IUGR rates [2]. In other studies, the increase in LBW rate was associated with maternal smoking, multiple births, increasing use of assisted reproductive technology, and changes in the perception of fetal viability [13-15].

In São Luís, Brazil, a second birth cohort was performed in 2010, and comparison with data from a previous birth cohort (undertaken in 1997/98) has shown that LBW rates remained stable, in contrast to the ascending rates that have been described earlier in the other two Brazilian cohorts performed in Pelotas [2] and Ribeirão Preto [12]. Since data from the two cohorts did not allow us to look at trends over time we also used registry data to verify if LBW rate really remained stable over that period in São Luís.

Thus, the objectives of the present study were to determine the temporal trend in LBW rate by analyzing historical series of birth registry data from the System of Information about Liveborns (SINASC in the Portuguese acronym) and to verify if risk factors for LBW and their prevalences differed between the two cohorts in the city of São Luís, Northeastern Brazil, comparing two birth cohorts separated by a 12-year interval. To avoid confounding by multiplicity, only singleton live births were included.

## Methods

Data was abstracted from two birth cohorts performed in the municipality of São Luís, the capital of Maranhão state, located in Northeastern Brazil, with a population of 1,014,837 inhabitants in 2010. São Luís has a human development index (HDI) of 0.768, the first in the state of Maranhão and the 249th in Brazil [16].

The first cohort included 2,831 hospital births from March 1997 to February 1998 and its methods have been published previously [5]. The second included 5,236 hospital births from the São Luís BRISA birth cohort project named “Etiologic factors of preterm birth and consequences of perinatal factors on children’s health: birth cohorts in two Brazilian cities”, which studied samples of births in the Brazilian cities of São Luís and Ribeirão Preto.

Data from the two birth cohorts are population based. Hospital births comprised 96.3% of all births in 1997/98 [5] and 98% in 2010. Maternity hospitals where less than 100 deliveries were performed were excluded from the study. This represented only 2.2% of the deliveries in 1997/98 and 3.3% in 2010. Thus, the sampling frame consisted of 94.1% of all births in 1997/98 and 94.7% in 2010.

The sample was stratified by maternity hospital with sharing proportional to the number of births at each facility. In each maternity hospital sampling was systematic and all live births and stillbirths were listed in order of occurrence. The sampling interval was seven in 1997/98 and three in 2010. A random number from 1 to 7 in 1997/98, and from 1 to 3 in 2010 was drawn to determine the starting point for each study unit. Thus one out of seven births in 1997/98 and one out of three births in 2010 were randomly selected for interview. Losses due to refusal or early discharge from hospital occurred in 5.8% of cases in 1997/98 and in 4.6% in 2010.

In the present study only data from mothers residing in São Luís, liveborns and singletons were used. In 1997/98, after exclusion of births from non-residents (n = 290), stillbirths (n = 48), multiples (n = 50) and missing information on study variables (n = 17), 2426 cases remained for analysis. In 2010, after exclusion of stillbirths (70), multiple births (99), newborns weighing <500 g (n = 3), and missing information on study variables (n = 24), data from 5,040 births remained for analysis.

Considering an 8.7% LBW rate for São Luís based on birth registry data, a 95% confidence level, and 1% absolute precision the necessary sample size would be 2,624 newborns.

The interviews were held within the first 24 hours after delivery by means of a standardized questionnaire including socioeconomic, demographic and life style questions; sexual and reproductive health; characteristics of current pregnancy and prenatal care; characteristics of delivery and birth. The newborns, wearing no clothing, were weighed on a baby scale with 5-gram graduations, shortly after delivery. The dependent variable was birth weight categorized as LBW (<2,500 g) and without LBW (≥2,500 g).

The independent variables were: newborn’s sex, maternal age (<18, 18-19, 20-34, ≥ 35 years), marital status (married, consensual union, with no companion), maternal schooling in years of study (0-4, 5-8, 9-11, ≥12), parity (1, 2, ≥3), maternal smoking during pregnancy (yes or no), monthly family income in minimum wages (≤1, 1-3, >3, missing), occupation of family head (unskilled manual worker, skilled manual worker, non-manual, missing), preterm birth (yes, < 37 or no, ≥ 37 weeks of gestation), type of delivery (vaginal or cesarean), and category of delivery care (public or private). Prenatal care utilization was classified as: none, inadequate, adequate, or missing data on gestational age and prenatal care. The adequacy of prenatal care use was measured using an index created on the basis of the minimum number of visits recommended by the Brazilian Health Ministry, and time of initiation. The index was adjusted for gestational age, because mothers of preterm babies tend to have fewer prenatal visits [17]. The categories showing the lowest LBW rate were used as reference.

The classification of IUGR by Kramer et al. [18] is based on the birth weight ratio (BWR), which was obtained by dividing the newborn’s birth weight by the median sex-specific weight for gestational age of the Canadian reference [19]. The NBs were considered to have no IUGR when BWR ≥0.85, to have mild IUGR when BWR ≥0.80 and <0.85, moderate IUGR when BWR ≥0.75 and <0.80, and severe IUGR when BWR < 0.75 [18]. In regression models IUGR was dichotomized into yes, when BWR < 0.85 and no otherwise.

In 1997 the monthly minimum wage was R$ 120.00, which corresponded to U$ 110.80 and in 2010 it was R$ 510.00, which corresponded to U$ 291.54 considering the value of the dollar on July 31^st^ of the respective years.

Gestational age (GA) was based on the date of the last menstrual period as reported by the mother. The 15^th^ day of the month was imputed for all cases in which the day of the last menstrual period was unknown. Birth weights located above the 99th percentile of the British reference [20], considered incompatible with GA, were recoded as missing. The same procedure was used in cases of improbable GA (less than 20 or more than 43 weeks). GA was imputed in a regression model based on birth weight, parity, per capita family income, and newborn’s sex. In 1997/98, 250 cases were imputed (10.3%), 7 as preterm and 243 as term births whereas in 2010 a total of 487 cases were imputed (9.7%), 29 of them as preterm births and 458 as term births. Gestational age in weeks was also classified into very preterm (<32), moderate preterm (32-33), late preterm (34-36), early term (37-38), full term (39-40), late term (41) and post term (≥42). Term births were classified following recommendations from the Defining “Term” Pregnancy Workgroup [21].

Birth registry data from SINASC (Portuguese acronym for the System of Information on Livebirths) for São Luís, from 1996 to 2010, were used to analyze trends in stillbirth and LBW rates, using 3-year moving averages. Births weighing <500 g, missing data on birth weight and multiples were excluded. The stillbirth rate was calculated as the ratio of the number of fetal deaths by the total number of births multiplied by 1,000.

The chi-square test was used to compare proportions between the two birth cohorts. Univariable analysis was performed using simple logistic regression to estimate non-adjusted odds ratios (OR) and their 95% confidence intervals (95%CI). Multivariable analysis was performed using multiple logistic regression. The independent variables that showed *p <* 0.20 were considered as candidates for the final model, but only those presenting p < 0.10 were retained. Data were analyzed using Stata 11.0.

Since LBW is due to preterm birth and IUGR, these variables were not selected for the multivariable model since they are mediators and they would tend to mask associations between maternal, socioeconomic and demographic factors with LBW.

Sensitivity analysis was conducted to examine the effect of imputation of gestational age on the findings reported. Selected data on maternal socioeconomic, demographic variables and birth weight from the two birth cohorts were compared with birth registry data by the chi-square test to assess how representative the cohorts are of the city births.

The project was approved by the Research Ethics Committee of the University Hospital, Federal University of Maranhão (protocol no.350/08 4771/2008-30) and all mothers gave written informed consent to participate before being interviewed.

## Results

**Figure 1:**
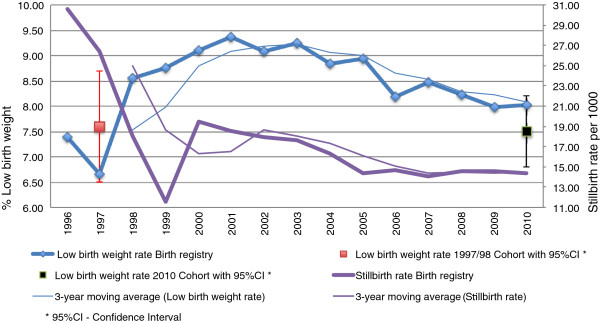
Low birth weight and stillbirth rates in São Luís from 1996 to 2010.

**Figure 2:**
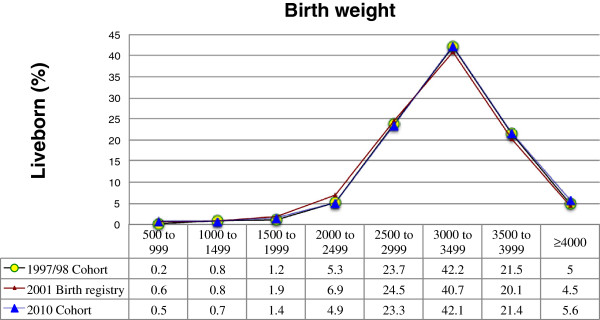
Birth weight distribution in the 1997/98 cohort, 2001 birth registry and 2010 cohort, São Luís, Brazil.

LBW rate among singletons was 7.6% in 1997/98 and 7.5% in 2010. There was no change in the low birth weight rate between the two cohorts (*p* = 0.847). However, observation of the historical series based on birth registry data from SINASC from 1996 to 2010 revealed variation between years, with an abrupt fall in 1997 and a later elevation from 1998 to 2001, when the rate peaked at 9.4%. The rate decreased thereafter up to 2009/2010 (Figure 1). This peak in LBW rate was observed in parallel with a leftward shift of the entire birth weight distribution due to higher births rates of NBs weighing 500-2,999 and to lower births rates of NBs weighing more than 3,000 g (Figure 2).

The stillbirth rate fell from 1996 to 1999, increased in 2000, fell until 2005, and stabilized thereafter (Figure 2).

**Figure 3:**
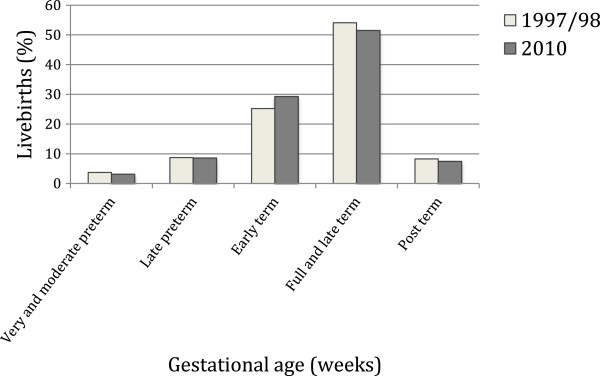
Distribution of livebirths according to gestational age, 1997/98 and 2010 São Luís birth cohorts.

**Figure 4:**
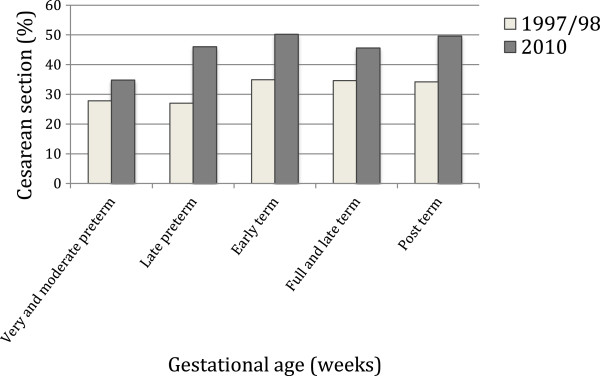
Distribution of cesarean section according to gestational age, 1997/98 and 2010 São Luís birth cohorts.

There was a slight decrease in the percentage of very and moderate preterm births from 1997/98 to 2010. The percentage of early term births increased from 25.2% to 29,3% whereas the percentage of full and late term and post term births decreased (Figure 3). Cesarean section rose in all gestational age groups but the increase was higher for late preterms (70% increase from 1997/98 to 2010), early terms (43.8% increase) and post terms (45% increase) (Figure 4).

Differences in NB characteristics were observed between 1997/98 and 2010. However, LBW, preterm birth and IUGR rates did not show statistically significant differences. In 1997/98 more boys were born (54.9%) than in 2010 (51.0%) (Table 1).

**Table 1:** Distribution of births according to the characteristics of the newborns, 1997/98 and 2010 São Luís birth cohorts

**Variables**	**São Luís 1997/98**		**São Luís 2010**		p*
	**n=2.426**		**n=5.040**		
	**n**	**%**	**n**	**%**	
**Newborn’s sex**					0.002
Male	1331	54.9	2569	51.0	
Female	1095	45.1	2471	49.0	
**Birth weight**					0.847
500-2,499	185	7.6	378	7.5	
≥2,500	2241	92.4	4662	92.5	
**Preterm birth**					0.424
Yes	301	12.4	593	11.8	
No	2125	87.6	4447	88.2	
IUGR^**^					0.394
No	2003	82.6	4183	83.0	
Mild	178	7.3	401	7.9	
Moderate	109	4.5	211	4.2	
Severe	136	5.6	245	4.9	

*p value calculated by the Pearson chi-square test.

**IUGR – intrauterine growth restriction based on the birth weight ratio.

Moreover, maternal, socioeconomic and demographic factors differed significantly between the two cohorts, except for smoking during pregnancy and parity. The percentage of teenage mothers (<20 years) was significantly reduced from 29.4% in 1997/98 to 18.7% in 2010. Conversely, there was a significant 80.9% increase in the percentage of mothers aged ≥35 years (*p* < 0.001). A significant improvement was observed in maternal schooling, with a 224.5% increase in the percentage of mothers with ≥12 years of study and a 380% reduction in the percentage of mothers with 0 to 4 years of study (*p* < 0.001). The proportion of mothers who were not living with a companion and of married mothers was reduced, while the percentage of mothers living in consensual union increased significantly (*p* < 0.001). There was a reduction in the percentage of families that lived on less than one minimum wage and of those living with >3 wages and an increase in the intermediate category (1-3 minimum wages) (*p* < 0.001). The results were worse regarding occupation of the family head, with a reduction of skilled manual workers and an increase in unskilled manual workers. Prenatal care use improved significantly, with an increase in the percentage of mothers who received adequate prenatal care and a reduction from 8.2% to 1.9% of those who received no prenatal care (*p* < 0.001). Cesarean section rate increased from 34.7% to 47.9% (*p* < 0.001). There was an increase in deliveries attended by private health care providers (*p* < 0.001) (Table 2).

**Table 2:** Distribution of births according to maternal, socioeconomic and demographic factors – 1997/98 and 2010 São Luís birth cohorts

**Variables**	**São Luís 1997/98**		**São Luís 2010**		p*
	**n= 2.426**		**n=5.040**		
	**n**	**%**	**n**	**%**	
**Maternal age (years)**					<0.001
< 18	317	13.1	437	8.7	
18 – 19	396	16.3	504	10.0	
20 – 34	1612	66.4	3713	73.7	
≥ 35	101	4.2	486	7.6	
**Maternal schooling (years)**					<0.001
0 - 4	416	17.1	227	4.5	
5 - 8	1031	42.5	1122	22.3	
9 - 11	860	35.5	2920	57.9	
≥12	119	4.9	758	11.0	
Missing	-	-	13	0.3	
**Marital status**					<0.001
Married	702	28.9	1101	21.8	
Consensual union	1137	46.9	2981	59.2	
Without a companion	587	24.2	958	19.0	
**Family income (minimum wages)**					<0.001
≤1	398	16.4	736	14.6	
1- 3	837	34.5	2040	40.5	
>3	1032	42.5	1362	27.0	
Missing	159	6.6	902	17.9	
**Maternal smoking during pregnancy**					0.212
No	2322	95.7	4854	96.3	
Yes	104	4.3	186	3.7	
**Occupation of the family head**					0.001
Non-manual	500	20.6	1036	20.5	
Skilled manual worker	1094	45.1	2050	40.7	
Unskilled manual worker	767	31.6	1789	35.5	
Missing	65	2.7	165	3.3	
**Parity**					0.627
1	1181	48.7	2404	47.7	
2	720	29.7	1549	30.7	
≥3	525	21.6	1087	21.6	
**Adequacy of prenatal care use**					<0.001
Adequate	1245	51.3	2988	59.3	
Inadequate	947	39.1	1700	33.7	
No care	199	8.2	95	1.9	
Missing	35	1.4	257	5.1	
**Category of delivery care**					<0.001
Private	268	11.0	801	15.9	
Public	2158	89.0	4239	84.1	
**Type of delivery**					<0.001
Vaginal	1608	66.3	2675	53.1	
Cesarean	818	34.7	2365	47.9	

*p-value calculated by the Pearson chi-square test.

In univariable analysis, preterm birth, IUGR, maternal age <18 years, primiparity, maternal smoking during pregnancy and cesarean section were risk factors for LBW in 1997/98 (Table 3). In 2010, preterm birth, IUGR, female sex, living without a companion, primiparity, maternal smoking during pregnancy and lack of prenatal care were risk factors for LBW. From 1997/98 to 2010 the OR for preterm birth increased from 10.42 to 20.92 (Table 4).

**Table 3:** Univariable analysis of risk factors for low birth weight, 1997/98 São Luís birth cohort

**Variables**	**% Low birth weight**	Odds ratio*	**95% Confidence interval**	p**
**Newborn’s sex**				0.701
Male	7.4	1	Reference	
Female	7.8	1.06	(0.78; 1.43)	
**Preterm birth**				<0.001
Yes	31.6	10.42	(7.55; 14.38)	
No	4.2	1	Reference	
IUGR***				<0.001
No	3.1	1	Reference	
Mild	5.1	1.66	(0.81; 3.41)	
Moderate	11.9	4.23	(2.25; 7.97)	
Severe	74.3	90.34	(57.02; 143.12)	
**Maternal age (years)**				0.005
<18	12.9	2.00	(1.37; 2.93)	
18 – 19	6.6	0.95	(0.61; 1.47)	
20 – 34	6.9	1	Reference	
≥35	6.9	1.00	(0.45; 2.22)	
**Maternal schooling (years)**				0.197
0 - 4	7.7	2.39	(0.82; 6.91)	
5 - 8	8.3	2.61	(0.94; 7.26)	
9 - 11	7.3	2.27	(0.81; 6.63)	
≥12	3.4	1	Reference	
**Marital status**				0.340
Married	6.4	1	Reference	
Consensual union	8.2	1.30	(0.89; 1.88)	
Without a companion	8.0	1.27	(0.83; 1.94)	
**Family income (minimum wages)**				0.073
≤1	9.3	1.25	(0.87; 1.78)	
1- 3	7.8	1.52	(1.00; 2.32)	
>3	6.3	1	Reference	
Missing	11.3	1.89	(1.09; 3.29)	
**Maternal smoking during pregnancy**				0.036
No	7.4	1	Reference	
Yes	13.5	1.95	(1.09; 3.50)	
**Occupation of the family head**				0.677
Non-manual	6.8	1	Reference	
Skilled manual worker	7.9	1.18	(0.78; 1.78)	
Unskilled manual worker	7.4	1.10	(0.70; 1.70)	
Missing	10.8	1.65	(0.70; 3.90)	
**Parity**				0.001
1	9.6	1.72	(1.19; 2.48)	
2	5.8	1	Reference	
≥3	5.5	0.94	(0.57; 1.53)	
**Adequacy of prenatal care use**				0.850
Adequate	7.9	1	Reference	
Inadequate	7.1	0.89	(0.64; 1.23)	
No care	8.5	1.09	(0.63; 1.87)	
Missing	8.6	1.09	(0.33; 3.64)	
**Category of delivery care**				0.133
Private	4.5	1	Reference	
Public	8.0	1.45	(0.87; 2.43)	
**Type of delivery**				0.027
Vaginal	7.5	1	Reference	
Cesarean	7.9	1.85	(1.02; 3.38)	

*Odds Ratio (OR) calculated by simple logistic regression.

**p-value calculated by the log likelihood ratio test.

***IUGR–intrauterine growth restriction based on the birth weight ratio.

**Table 4:** Univariable analysis of risk factors for low birth weight, 2010 São Luís birth cohort

**Variables**	**%Low birth weight**	Odds ratio*	**95% Confidence interval**	p**
**Newborn’s sex**				0.026
Male	6.7	1	Reference	
Female	8.3	1.26	(1.02; 1.56)	
**Preterm birth**				<0.001
Yes	40.3	20.92	(16.53; 26.48)	
No	3.1	1	Reference	
IUGR***				<0.001
No	3.3	1	Reference	
Mild	8.0	2.56	(1.97; 4.05)	
Moderate	15.2	5.27	(3.79; 8.13)	
Severe	72.2	76.87	(55.40; 106.64)	
**Maternal age(years)**				0.107
<18	9.4	1.38	(0.98; 1.95)	
18 – 19	8.7	1.28	(0.91; 1.78)	
20 – 34	6.9	1	Referência	
≥35	9.1	1.33	(0.92; 1.93)	
**Maternal schooling (years)**				0.861
0 to 4	7.0	0.96	(0.54; 1.72)	
5 to 8	7.2	0.99	(0.66; 1.41)	
9 to 11	7.7	1.06	(0.78; 1.44)	
≥12	7.3	1	Reference	
Missing	15.4	2.32	(0.50; 10.74)	
**Marital status**				0.020
Married	6.3	1	Reference	
Consensual union	7.3	1.18	(0.89; 1.56)	
Without a companion	9.5	1.56	(1.13; 2.17)	
**Family income (minimum wages)**				0.107
≤1	8.3	1.37	(0.97; 1.93)	
1- 3	7.9	1.31	(0.99; 1.72)	
>3	6.2	1	Reference	
Missing	7.8	1.29	(0.93; 1.80)	
**Maternal smoking during pregnancy**				<0.001
No	7.2	1	Reference	
Yes	16.1	2.49	(1.65; 3.73)	
**Occupation of the family head**				0.346
Non-manual	6.9	1	Reference	
Skilled manual worker	8.2	1.20	(0.90; 1.60)	
Unskilled manual worker	6.9	0.98	(0.73; 1.33)	
Missing	8.5	1.24	(0.68; 2.25)	
**Parity**				0.003
1	8.8	1.50	(1.16; 1.93)	
2	6.0	1	Reference	
≥3	6.8	1.14	(0.83; 1.56)	
**Adequacy of prenatal care use**				<0.001
Adequate	7.6	1	Reference	
Inadequate	6.8	0.89	(0.70; 1.12)	
No care	20.0	3.04	(1.80; 5.11)	
Missing	6.2	0.80	(0.47; 1.36)	
**Category of delivery care**				0.293
Private	6.6	1	Reference	
Public	7.7	1.17	(0.86; 1.58)	
**Type of delivery**				0.265
Vaginal	7.9	1	Reference	
Cesarean	7.1	0.88	(0.71; 1.09)	

*Odds Ratio (OR) calculated by simple logistic regression.

**p-value calculated by the log likelihood ratio test.

***IUGR–intrauterine growth restriction based on the birth weight ratio.

The variables that were significantly associated with LBW in the final model in the 1997/98 cohort were maternal smoking during pregnancy (OR = 2.30) and primiparity (OR = 1.66). The variables that remained in the adjusted model for the 2010 cohort were female sex (OR = 1.29), maternal age ≥35 years (OR = 1.55), family income from 1 to 3 minimum wages (OR = 1.38), lack of prenatal care (OR = 2.79), maternal smoking during pregnancy (OR = 2.51), and primiparity (OR = 1.57) (Table 5).

**Table 5:** Multivariable analysis of risk factors for LBW, 1997/98 and 2010 São Luís birth cohorts

	**1997/98 Cohort**			**2010 Cohort**		
**Variable**	Odds ratio*	**95% Confidence interval**	p*	Odds ratio*	**95% Confidence interval**	p**
**Maternal age (years)**			0.082			0.142
<18	1.51	(0.99; 2.29)		1.10	(0.75; 1.59)	
18 – 19	0.78	(0.49; 1.24)		1.09	(0.77; 1.55)	
20 – 34	1	Reference		1	Reference	
≥35	1.19	(0.52; 2.70)		1.55	(1.06; 2.27)	
**Newborn’s sex**			0.686			0.017
Male	1	Reference		1	Reference	
Female	1.06	(0.78; 1.44)		1.29	(1.04; 1.59)	
**Family income**			0.107			0.119
≤1	1.52	(0.97; 2.37)		1.41	(0.99; 2.03)	
1- 3	1.21	(0.83; 1.76)		1.38	(1.04; 1.84)	
>3	1	Reference		1	Reference	
Missing	1.82	(1.03; 3.22)		1.31	(0.92; 1.84)	
**Adequacy of prenatal care use**			0.455			<0.001
Adequate	1	Reference		1	Reference	
Inadequate	0.76	(0.53; 1.07)		0.86	(0.67; 1.10)	
No care	0.98	(0.55; 1.77)		2.79	(1.60; 4.86)	
Missing	0.90	(0.26; 3.10)		0.78	(0.46; 1.34)	
**Maternal smoking during pregnancy**			0.007			<0.001
No	1	Reference		1	Reference	
Yes	2.30	(1.25; 4.23)		2.51	(1.63; 3.84)	
**Parity**			0.003			<0.001
1	1.66	(1.13; 2.43)		1.57	(1.20; 2.05)	
2	1	Reference		1	Reference	
≥3	0.83	(0.50; 1.38)		1.10	(0.53; 1.77)	
**Category of delivery care**			0.050			0.916
Private	1	Reference		1	Reference	
Public	1.94	(0.99; 3.78)		0.97	(0.58; 1.60)	
**Type of delivery**			0.064			0.403
Vaginal	1	Reference		1	Reference	
Cesarean	1.38	(0.98; 1.95)		1.12	(0.82; 1.53)	

*Odds Ratio (OR) calculated by multiple logistic regression.

**p-value calculated by the log likelihood ratio test.

In 1997, cohort data did not differ from birth registry data regarding birth weight, maternal age and type of delivery. Males were slightly overrepresented in the cohort compared to females (p = 0.010). Although there were differences with respect to maternal schooling, missing data were too high in birth registry data for a meaningful comparison (Additional file 1: Table S1). It was not possible to compare marital status in 1997, because this information was not available in the birth registry. In 2010 newborn’s sex, birth weight and maternal age did not differ significantly comparing birth registry with cohort data. Low schooling, married mothers and those born by cesarean delivery were slightly underrepresented in the 2010 cohort (Additional file 1: Table S2).

Sensitivity analysis was performed and the imputation did not affect the findings (results now shown, available on request).

## Discussion

A significant improvement in access to prenatal care and in maternal schooling was observed, as well as a reduction in the percentage of teenage mothers, a fall in the stillbirth rate and an increase in deliveries held at private facilities. However, LBW rate was not significantly different comparing data from the two birth cohorts at the two time points.

In contrast, analysis of birth registry data from SINASC revealed that the stability in the LBW rate observed between the two cohorts actually masked an abrupt fall in 1997 and an increase from 1998 to 2001, with a later reduction up to 2010.

It is worth noting that LBW rate increased whereas stillbirth rates decreased from 1996 to 2001. From 2001 onwards, LBW and stillbirth rates tended to fall simultaneously. According to data from the two birth cohorts, stillbirth rate decreased from 18.9 in 1997/98 to 13.4 per thousand in 2010. These changes suggest improving obstetric and neonatal care including careful fetal surveillance and prompt obstetric intervention where indicated [22]. From 1996 to 2001 low birth weight fetuses instead of dying in utero (stillbirths) were rescued and born alive, thus increasing LBW rate. Later on possibly obstetric and neonatal care improved further still leading to simultaneously decreasing stillbirth and LBW rates.

There was a significant reduction in the percentage of mothers who did not receive prenatal care in the 12-year interval. The absence of prenatal care was considered to be a risk factor for LBW only in 2010. With increasing access to prenatal care, there was a probable increase in the contrast between the better off and more vulnerable groups who showed the worst outcomes. According to some studies [7,20,23,24], the effect of the number of prenatal visits on the reduction of LBW rate is doubtful. However, prenatal care has advantages at the individual level, suggesting that it is necessary to investigate the content of the care offered in order to identify the more effective components and the best way to structure prenatal care [7].

Extremes of maternal age were associated with LBW, with differences between cohorts. In 1997/98 an association was observed with maternal age of more than 18 years, whereas in 2010 age ≥35 years was associated with higher LBW rate. The association between more advanced age in 2010 was not detected in 1997/98, possibly due to the increased proportion of women who, more recently, have been engaged in conquering their space on the job market and having financial stability, a fact that delays maternity plans. However, a fall in fertility occurs with advancing age, a fact that often leads women to seek ovarian stimulation and assisted reproductive technology, which represent two major risk factors for twinning and LBW [25].

Maternity at more advanced ages seem to be a more serious public health problem than teenage pregnancy. The prognosis of pregnancy in women older than 35 years is worse than that for teenage mothers. Pregnancy during the age range of highest risk can be prevented with public policies supporting women who hold jobs outside home.

An improvement in socioeconomic level was observed based on maternal schooling, the reduction of families who live on less than one minimum wage, and the increased proportion of families with an income of 1-3 minimum wages. Conversely, proportion of non-skilled manual workers rose and the percentage of families earning more than 3 minimum wages was reduced, what suggests worsening in life conditions. A possible explanation for this apparent contradiction is the increase in the monthly minimum wage, which went from U$ 110.80 to U$ 291.54 during this period. Thus, the fall in the proportion of persons earning more than 3 minimum wages did not represent a worse mean family income but reflected the increased purchasing power of the minimum wage during this period.

Among the socioeconomic variables analyzed, only income of less than 3 minimum wages was associated with LBW in 2010, while the remaining variables showed no statistical significance. In a study conducted in the city of São Paulo, the authors pointed out the low magnitude of the difference detected between the various social strata with respect to LBW [26]. Another study conducted in Ribeirão Preto based on two cohorts (1978/79 and 1994) revealed an association between income of less than 4 minimum wages and LBW in the first cohort, in 1978/79. However, in the second cohort, in 1994 the authors observed an increase in the LBW rate among children of mothers who held executive and academic jobs, a fact that may have contributed to the disappearance of the association of income with LBW in this case [12]. In the city of Pelotas, in 1982 and 1993, family income of less than one minimum wage per month was associated with LBW. However, in 2004 this scenario changed, with increasing LBW rate observed among newborns from families of higher income [2]. A study conducted in Rio de Janeiro demonstrated that there were social inequalities according to LBW, as measured by income of the family head and maternal schooling, with income being the more sensitive indicator [27].

Marital status was significantly associated with LBW only in univariable analysis in the 2010 cohort, with a loss of significance after adjustment. Following changes in the way of life and in thinking of today’s society, there was a reduction of prejudice regarding single mothers. Single mothers possibly started to count on greater social support, with a reduction of stress. From this perspective, the studies are inconclusive regarding the relation between marital status and LBW [1].

More girls started to be born in 2010, but the percentage of boys continued to be higher. Newborn’s sex was not associated with LBW in the 1997/98 cohort, but in 2010 female sex was associated with 1.26 increased risk of LBW. Other studies have also shown association between newborn’s sex and LBW, demonstrating that females have a higher risk of being born weighing less than 2,500 g [12,28].

There was no change in the proportion of mothers who smoked during pregnancy. Maternal smoking was associated with LBW in both cohorts, in agreement with other reports [12,26].

Primiparity was associated with LBW in both cohorts studied, in accordance with a synopsis based on scientific evidence about LBW [7].

The two highest ORs for LBW in 2010 are for no prenatal care and for smoking. It is very important to continue improving access to prenatal care and promoting smoking cessation to reduce LBW rate.

Despite the significant increase in cesarean section rate, type of delivery was not associated with LBW in the adjusted analysis. This association was only observed in univariable analysis in 1997/98. After adjustment for confounding variables, type of delivery was not considered to be a risk factor for LBW in any of the cohorts studied, in contrast to other studies [2,12]. Previous studies conducted in Brazil have shown that incorrect late ultrasound dating of gestational age was associated with increased iatrogenic preterm births [2,29]. This lack of association between cesarean section and LBW in the later cohort may be explained in part by greater access to early ultrasound exams that provide a better dating of gestational age. Thus, although many unnecessary cesarean deliveries were still performed because of convenience (cesarean section rate was very much higher than the maximum recommended rate for medical reasons [30]), cesarean section was not associated with iatrogenic prematurity and LBW in the 2010 cohort. An association between cesarean section and LBW has been reported in a previous study using data from the earlier cohort (1997/98) [31]. However, there was an increase in early term births, what might be associated with increasing cesarean section rates from 1997/98 to 2010. Increasing early term births has been associated with medical intervention and has deleterious effect on subsequent offspring’s health and development [32,33].

The strengths of the present study are that data were abstracted from two population based cohort studies conducted on large samples, with low percentage of losses. Validated measurements were used. Under-registration of birth weights in registry data from SINASC, especially in 1997, represents a limitation. Another limitation is that there was slightly overrepresentation of male births in the 1997/98 cohort and underrepresentation of low schooling and married mothers and those born by a cesarean section. However, there are some concerns regarding quality of registry data, especially in the earlier period [34]. Lack of information on maternal body mass index, maternal race/ethnicity and mother’s diseases for the 1997/98 birth cohort prevented us from using these variables in this study, since our objective was to compare risk factors for LBW in the two birth cohorts.

In addition there was a high percentage of missing results regarding gestational age. However, this limitation was partially remedied by imputation of gestational age. Furthermore, results regarding preterm birth and intrauterine growth restriction rates did not change using unimputed gestational age data.

## Conclusions

Despite improved living conditions regarding maternal schooling, prenatal care use, and childbirth care, no change was observed in birth weight distribution comparing the two birth cohorts. However, this apparent stability masked an increase in LBW rate from1996 to 2001. The increase and the fall in LBW paralleled reduction in stillbirths, a fact that suggests improving obstetrical and neonatal care. Maternal, socioeconomic and demographic factors associated with LBW differed significantly between the two cohorts, except for smoking during pregnancy and parity that were significantly associated with LBW in both cohorts.

## Abbreviations

LBW: Low birth weight; OR: Odds ratio; IUGR: Intrauterine growth restriction; NB: Newborn; SINASC: Portuguese acronym for the Information System on liveborns.

## Competing interests

The authors declare that they have no competing interests.

## Authors’ contribution

HJFV and AAMdS contributed to the conception of the project, the analysis and interpretation of the data and the writing of the paper and analyzed the article for final approval of the version to be published. HB, MZG, FLF, VMFS, RFLB and MAB contributed to the analysis and interpretation of the data and the writing of the paper and performed a relevant critical review of the manuscript intellectual content and analyzed the article for final approval of the version to be published.

## Pre-publication history

The pre-publication history for this paper can be accessed here:

http://www.biomedcentral.com/1471-2393/14/155/prepub

## Supplementary Material

Additional file 1: Table S1 Comparison of socioeconomic, demographic characteristics and birth weight between birth registry and birth cohort data, São Luís, 1997. **Table S2.** Comparison of socioeconomic, demographic characteristics and birth weight between birth registry and birth cohort data, São Luís, 2010. Click here for file
