# Supplementary material for: Low birth weight in São Luís, northeastern Brazil: trends and associated factors
Source: BMC Pregnancy Childbirth. 2014 May 1;14:155. doi: 10.1186/1471-2393-14-155 (PMC4108005; doi:10.1186/1471-2393-14-155)
Supplement: Additional file 1: Table S1 — Comparison of socioeconomic, demographic characteristics and birth weight between birth registry and birth cohort data, São Luís, 1997. Table S2. Comparison of socioeconomic, demographic characteristics and birth weight between birth registry and birth cohort data, São Luís, 2010. [file 1471-2393-14-155-S1.doc]

Additional file 1: Table S1. Comparison of socioeconomic, demographic characteristics and birth weight between birth registry and birth cohort data, São Luís, 1997.

| **Variables** | **Cohort data**  **n= 2426** | | **Registry data from SINASC***  **n=15074** | | ***p***** |
| --- | --- | --- | --- | --- | --- |
|  | n | % | n | % |  |
| **Newborn’s sex** |  |  |  |  | 0.010 |
| Male | 1331 | 54.9 | 7820 | 52.1 |  |
| Female | 1095 | 45.1 | 7201 | 47.9 |  |
| Missing | - | - | 53 | - |  |
| **Birth weight** |  |  |  |  | 0.078 |
| 500-2499 | 185 | 7.6 | 986 | 6.7 |  |
| ≥2500 | 2241 | 92.4 | 13828 | 93.3 |  |
| Missing | - | - | 260 | - |  |
| **Maternal age (years)** |  |  |  |  | 0.251 |
| < 20 | 713 | 29.4 | 3891 | 28.2 |  |
| 20 – 29 | 1413 | 58.2 | 8012 | 58.1 |  |
| 30 – 39 | 284 | 11.7 | 1785 | 12.9 |  |
| ≥ 40 | 16 | 0.7 | 112 | 0.8 |  |
| Missing | - | - | 1274 | - |  |
| **Maternal schooling (years)** |  |  |  |  | <0.001 |
| 0 - 8 | 1447 | 59.6 | 8100 | 64,7 |  |
| 9 - 11 | 860 | 35.5 | 3932 | 31,4 |  |
| ≥ 12 | 119 | 4.9 | 497 | 3,9 |  |
| Missing | - | - | 2545 | - |  |
| **Marital status** |  |  |  |  |  |
| Married | 702 | 28.9 | - | - |  |
| Consensual union | 1137 | 46.9 | - | - |  |
| Without a companion | 587 | 24.2 | - | - |  |
| Missing | - | - | 15074 | 100 |  |
| **Type of delivery** |  |  |  |  | 0.328 |
| Vaginal | 1608 | 66.3 | 9796 | 65.3 |  |
| Cesarean | 818 | 33.7 | 5214 | 34.7 |  |
| Missing | - | - | 64 | - |  |

***SINASC – Portuguese acronym for the Brazilian birth registry – System of Information on Live births

**p value calculated by the Pearson chi-square test excluding missing values

Additional file 1: Table S2. Comparison of socioeconomic, demographic characteristics and birth weight between birth registry and birth cohort data, São Luís, 2010.

| **Variables** | **Cohort data**  **n=5040** | | **Registry data from SINASC**  **n=16861** | | ***p**** |
| --- | --- | --- | --- | --- | --- |
|  | **n** | **%** | **n** | **%** |  |
| **Newborn’s sex** |  |  |  |  | 0.725 |
| Male | 2569 | 51.0 | 8641 | 51.3 |  |
| Female | 2471 | 49.0 | 8218 | 48.7 |  |
| Missing | - | - | 2 | - |  |
| **Birth weight** |  |  |  |  | 0.220 |
| 500-2499 | 378 | 7.5 | 1354 | 8.0 |  |
| ≥2500 | 4662 | 92.5 | 15506 | 92.0 |  |
| Missing | - | - | 1 | - |  |
| **Maternal age (years)** |  |  |  |  | 0.198 |
| <20 | 941 | 18.7 | 2961 | 17.6 |  |
| 20 – 29 | 2939 | 58.3 | 9851 | 58.4 |  |
| 30 – 39 | 1078 | 21.4 | 3783 | 22.4 |  |
| ≥40 | 82 | 1.6 | 266 | 1.6 |  |
| **Maternal schooling (years)** |  |  |  |  | <0.001 |
| 0 - 3 | 143 | 2.8 | 370 | 2.3 |  |
| 4 - 7 | 641 | 12.8 | 3028 | 18.8 |  |
| 8 - 11 | 3485 | 69.3 | 9516 | 59.2 |  |
| ≥12 | 758 | 15.1 | 3155 | 19.6 |  |
| Missing | 13 | - | 792 | - |  |
| **Marital status** |  |  |  |  | 0.003 |
| Married | 1101 | 21.8 | 3929 | 23.9 |  |
| Not married | 3939 | 78.2 | 12536 | 76.1 |  |
| Missing | - | - | 396 | - |  |
| **Type of delivery** |  |  |  |  | 0.003 |
| Vaginal | 2675 | 53.1 | 8503 | 50.7 |  |
| Cesarean | 2365 | 46.9 | 8282 | 49.3 |  |
| Missing | - | - | 76 | - |  |

***SINASC – Portuguese acronym for the Brazilian birth registry – System of Information on Live births

**p value calculated by the Pearson chi-square test excluding missing values
